# The gut microbiota metabolite phenylacetylglycine regulates cardiac Ca^2+^ signaling by interacting with adrenergic receptors

**DOI:** 10.21203/rs.3.rs-6701722/v1

**Published:** 2025-06-12

**Authors:** Elisa Bovo, Aleksey V. Zima

**Affiliations:** Department of Cell & Molecular Physiology, Stritch School of Medicine, Loyola University Chicago, Maywood, IL, 60153 USA

**Keywords:** heart, Ca2+ signaling, adrenergic receptor, gut microbiota, confocal microscopy

## Abstract

Phenylacetylglutamine (PAGln) and phenylacetylglycine (PAGly) are small molecules derived from the metabolism of phenylalanine by gut microbiota. Elevated levels of PAGln and PAGly in serum have been associated with increased risks for cardiovascular diseases. It has been suggested that PAGln and PAGly reduce cardiac contraction by blunting the adrenergic response during sympathetic stimulation. However, little is known whether the effect of PAGln and PAGly on the heart function is associated with an alteration of intracellular Ca^2+^ homeostasis. Here, we studied the effect of PAGly on Ca^2+^ regulation in mouse ventricular myocytes, as PAGly is the predominant phenylalanine metabolite in rodent’s serum. Analysis of cytosolic Ca^2+^ dynamics revealed that PAGly (100 μM) increases action potential-induced Ca^2+^ transients and sarcoplasmic reticulum (SR) Ca^2+^ load. These effects of PAGly were significantly smaller than those produced by the adrenergic receptor agonist isoproterenol (ISO; 0.1 μM). The adrenergic receptor blocker propranolol (10 μM) and the protein kinase A (PKA) inhibitor H89 (10 μM) prevented the PAGly effects on intracellular Ca^2+^ dynamics. Further analysis of Ca^2+^ regulation revealed that pretreatment of cardiomyocytes with PAGly reduced the stimulatory effect of ISO on intracellular Ca^2+^ dynamics. Concurrently, PAGly did not produce any stimulatory effects on intracellular Ca^2+^ in the presence of ISO. In conclusion, PAGly regulates intracellular Ca^2+^ dynamics in ventricular myocytes by activating the adrenergic receptor-mediated signaling, but less efficiently than selective adrenergic agonists. By interacting with adrenergic receptors, PAGly can partially blunt the stimulatory effect of adrenergic receptor agonists.

## INTRODUCTION

Heart failure remains the leading cause of morbidity and mortality, affecting more than 64 million people worldwide [1]. The search for new therapeutic approaches to improve the prognosis of heart diseases has recently revealed a potential link between gut microbiome metabolites and cardiac function. Metabolomic analysis uncovered a positive correlation between elevated levels of plasma phenylacetylglutamine (PAGln) and phenylacetylglycine (PAGly) and increased risk for cardiovascular diseases [2–4]. PAGln and PAGly are small molecules produced by the gut microbiota during metabolism of unabsorbed phenylalanine. In brief, bacteria in the gut convert phenylalanine into phenylacetic acid, which can be conjugated in liver with the amino acid glutamine to form PAGln or with glycine to form PAGly [5]. Previous analysis revealed that PAGln is the main metabolite of phenylalanine in human, whereas PAGly is more abundant in rodents [6]. It has been suggested that one of the mechanisms through which PAGln and PAGly can affect the cardiovascular system is by binding to G protein-coupled adrenergic receptors [6]. Furthermore, functional studies of mouse cardiac function showed that PAGly can attenuate the positive inotropic effect of adrenergic receptor stimulation [7]. However, specific mechanisms by which phenylalanine metabolites affect heart function have not been fully understood.

Regular contraction and relaxation of the heart are governed by tightly controlled intracellular Ca^2+^ cycling. The majority of Ca^2+^ that activates contraction during systole is released from the sarcoplasmic reticulum (SR) via the type 2 ryanodine receptor (RyR2). During the action potential, a relatively small inward Ca^2+^ current via the L-type Ca^2+^ channels (LTCC) activates RyR2 by a mechanism called Ca^2+^-induced Ca^2+^ release (CICR) [8]. After termination of CICR, diastolic relaxation occurs when excessive Ca^2+^ is removed from the cytosol by the sarco(endo)plasmic reticulum Ca^2+^-ATPase (SERCA2a) and by the Na^+^/Ca^2+^ exchanger (NCX). One of key mechanisms that regulates intracellular Ca^2+^ dynamics and heart contraction is mediated by β-adrenergic receptor activation during sympathetic stimulation. Stimulation of β-adrenergic receptors activates adenylyl cyclase and increases cytosolic cAMP level, which in turn activates protein kinase A (PKA) [9]. Several key proteins involved in regulation of Ca^2+^ homeostasis are targets of PKA-mediated phosphorylation, including the SERCA2a regulator phospholamban (PLB), RyR2 and LTCC [10]. PKA-dependent phosphorylation results in an increase in activity of Ca^2+^ pumps and Ca^2+^ channels, causing more synchronized and robust myocardial contraction during systole and faster relaxation during diastole[9,11]. Such complex response to β-adrenergic receptor activation guarantees increased cardiac output during physical and emotional stress. Consequently, abnormal adrenergic signaling can lead to Ca^2+^ mishandling and contractile dysfunctions, commonly seen during the development of heart failure [9].

The aim of this study was to determine mechanisms by which PAGly affects intracellular Ca^2+^ dynamics in mouse ventricular myocytes. This study revealed that PAGly can produce a mild stimulatory effect on Ca^2+^ dynamics, and this effect is predominantly mediated by β-adrenergic receptor and PKA activation. By competing for binding to β-adrenergic receptors, PAGly can partially blunt the stimulatory effect of more selective adrenergic receptor agonists.

## MATERIALS and METHODS

### Isolation of ventricular myocytes.

All animal husbandry and surgical procedures were carried out in accordance with the National Institutes of Health guide for the care and use of Laboratory animals [12]. Procedures are covered by Loyola IACUC protocols #23–027 (mice). C57BL/6J mice (8 of male and 5 of female animals, Jackson Laboratories) aged between 2–3 months were anesthetized using Isoflurane (1%). Following thoracotomy, hearts were quickly excised, immersed in Ca^2+^ free buffer, mounted on a Langendorff apparatus, and retrogradely perfused with a solution (37°C) containing Liberase H (Roche Sigma-Aldrich; St Louis, MO, USA), according to a procedure described previously [13–15]. The left ventricle was excised from the digested heart, placed in stop buffer containing BSA 1 mg/mL, cut into several pieces (average size 1 mm) and gently triturated into a suspension of isolated myocytes. Then, myocytes were pelleted by gravity (~0.1 ml) and resuspended in low-Ca^2+^ Tyrode buffer (in mM: NaCl 140; KCl 4; CaCl_2_ 0.1; MgCl_2_ 1; glucose 10; Hepes 10; pH 7.4). [Ca^2+^] was gradually adjusted to 1 mM. Isolated cardiomyocytes were stored at room temperature (20°C) during an experiment. All chemicals and reagents were purchased from Sigma-Aldrich.

### Confocal Microscopy and Ca^2+^ imaging.

Changes in the cytosolic [Ca^2+^] ([Ca^2+^]_i_) were measured with laser scanning confocal microscopy (Radiance 2000 MP, Bio-Rad, UK) equipped with a ×40 oil-immersion objective lens (N.A.=1.3). Changes in [Ca^2+^]_i_ were recorded with the high-affinity Ca^2+^ indicator Fluo-4 (Molecular Probes/Invitrogen, Carlsbad, CA, USA). To load the cytosol with Fluo-4, ventricular myocytes were incubated at room temperature with 10 μM Fluo-4 AM for 15 min in Tyrode solution (in mM: NaCl 140; KCl 4; CaCl_2_ 2; MgCl_2_ 1; glucose 10; Hepes 10; pH 7.4), followed by a 20 min wash. Fluo-4 was excited with the 488 nm line of an argon ion laser and fluorescence was measured at wavelengths > 515 nm. Images were acquired in the line-scan mode (4 ms per scan; pixel size 0.3 μm). The scan line was oriented parallel to the longitudinal axis of the cell avoiding the nucleus. Whole-cell [Ca^2+^]_i_ transients were obtained by averaging the entire cellular fluo-4 signal from the scanned line. [Ca^2+^]_i_ transients are presented as background-subtracted normalized fluorescence (F/F_0_) where F is the fluorescence intensity and F_0_ is resting fluorescence recorded under steady-state conditions at the beginning of an experiment. [Ca^2+^]_i_ transients were evoked by electrical field stimulation with 2 ms suprathreshold voltage pulses applied through a pair of extracellular platinum electrodes at a frequency of 0.5 Hz.

### Statistics.

Data are presented as mean ± standard error of the mean (SEM) of n measurements. Statistical comparisons between groups were performed using Student’s t test for unpaired data sets. Differences were considered statistically significant at P<0.05. Statistical analysis and graphical representation of average data was carried out using the OriginPro7.5 software (OriginLab, USA).

## RESULTS

### Effect of PAGly on intracellular Ca^2+^ signaling in mouse ventricular myocytes.

**Figure 1:**
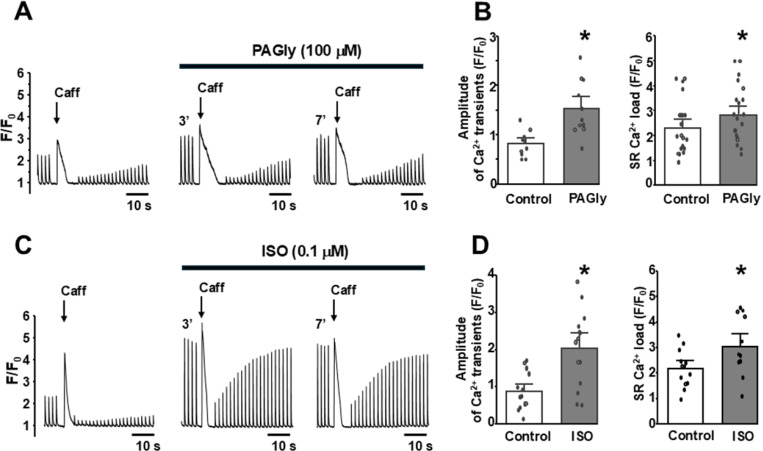
Effect of PAGly and ISO on Ca^2+^ signaling in ventricular myocytes. **A,** F/F_0_ profiles of cytosolic Ca^2+^ during AP-induced and caffeine-induced Ca^2+^ transients (or SR Ca^2+^ load) in control conditions and during PAGly (100 mM) application. **C,** changes in cytosolic Ca^2+^ during AP-induced Ca^2+^ transients and SR Ca^2+^ load in control conditions and during ISO (0.1 mM) application. The average effects of PAGly (**B,** n = 19 myocytes) and ISO (**D,** n = 11 myocytes) on AP-induced Ca^2+^ transient amplitude and SR Ca^2+^ load. *P<0.05 vs Control.

**Figure 2:**
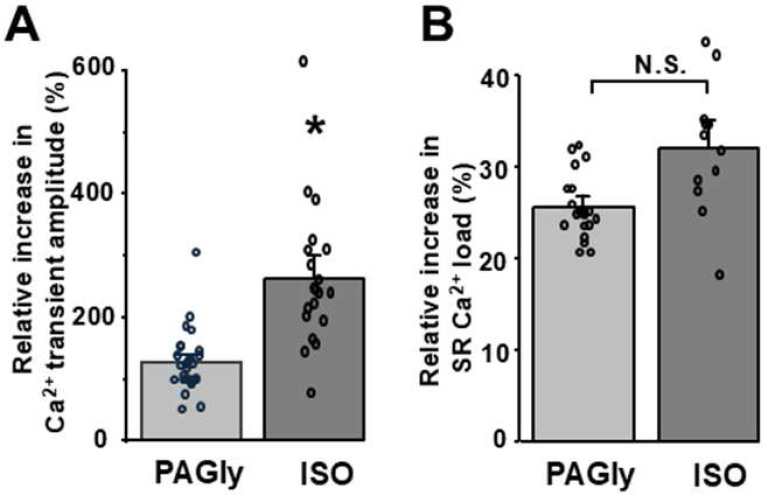
Relative effect of PAGly and ISO on AP-induced Ca^2+^ transients and SR Ca^2+^ load. **A,** a relative increase of AP-induced Ca^2+^ transients induced by PAGly (100 mM) and ISO (0.1 mM). **B,** a relative increase of SR Ca^2+^ load induced by PAGly (100 mM) and ISO (0.1 mM). *P<0.05 vs Control.

**Figure 3:**
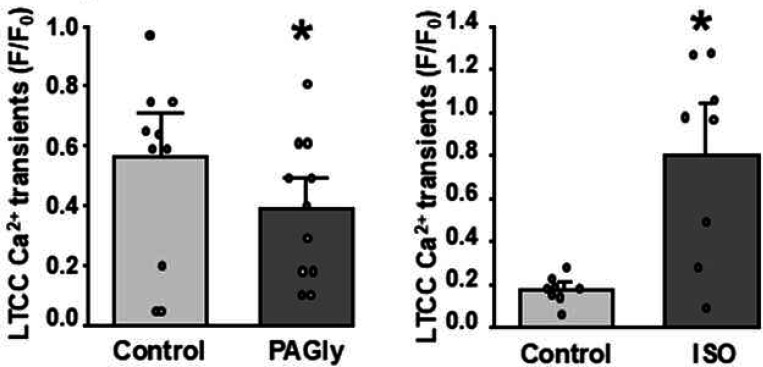
Effect of PAGly and ISO on LTCC-induced Ca^2+^ transients in ventricular myocytes. Amplitude of the first AP-induced Ca^2+^ transient after complete [Ca^2+^]_SR_ depletion with caffeine (Fig 1 A and C) was used as an index of the inward Ca^2+^ current amplitude via LTCC.

To assess the effect of PAGly on intracellular Ca^2+^ signaling, action potential (AP)**-** and caffeine**-**induced Ca^2+^ transients were measured in mouse ventricular myocytes (Fig. 1A). Myocytes were electrically stimulated (0.5 Hz) to evoke regular cytosolic Ca^2+^ transients induced by APs. Then electrical stimulation was suspended for 5 s and caffeine (Caff; 5 mM) was applied to activate RyR2, which resulted in the release of all Ca^2+^ stored within the SR. The amplitude of SR Ca^2+^ release during caffeine application was used as an index of SR Ca^2+^ load. PAGly (100 pM) increased AP-induced Ca^2+^ transients to 200±12% (n=19 cells; Fig. 1B, left) and SR Ca^2+^ load to 124±7% (n=19 cells; Fig. 1B, right) compared to 100% in control. Furthermore, PAGly increased the fraction of SR Ca^2+^ released during APs (fractional release = AP Ca^2+^ transient/SR Ca^2+^ load) to 125±8% (n=19 cells**Error! Reference source not found.**). Next, we compared the effect of PAGly on Ca^2+^ dynamics with the effect that produces the P-adrenergic receptor agonist ISO (0.1 μM; Fig 1C). ISO (0.1 μM) increased the AP-induced Ca^2+^ transient amplitude to 270±28% (n=18 cells; Fig. 1D, left) and SR Ca^2+^ load to 133±35% (n=11 cells; Fig. 1D, right) compared to 100% in control. Overall, the increase in AP-induced Ca^2+^ transients produced by PAGly was significantly smaller than those induced by ISO (Fig. 2, A). However, both PAGly and ISO produced a relatively similar increase in SR Ca^2+^ load (Fig. 2, B). The first AP-induced Ca^2+^ transient after complete [Ca^2+^]_SR_ depletion with caffeine is mainly mediated by inward Ca^2+^ current via LTCC [16]. Analysis of LTCC-mediated Ca^2+^ transients revealed that PAGly decreased these transients to 70±15% (n=19 cells; Fig. 3, A), whereas ISO increased LTCC-mediated Ca^2+^ transients to 377±115% (n=11 cells; Fig. 3, B).

### Role of β-adrenergic receptor and PKA activation in the PAGly effect on Ca^2+^ signaling.

**Figure 4:**
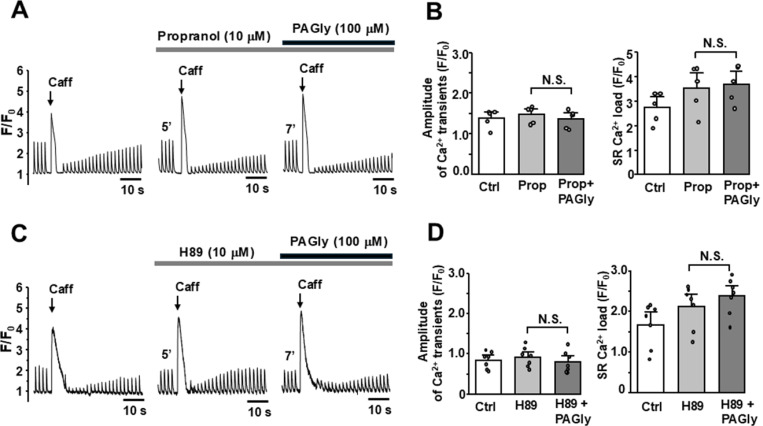
Effect of PAGly on Ca^2+^ signaling during adrenergic receptor or PKA inhibition. **A,** F/F_0_ profiles of cytosolic Ca^2+^ during AP- and caffeine-induced Ca^2+^ transients in control conditions, during adrenergic receptor inhibition with propranolol (Pro; 0.1 mM) with following application of PAGly (100 mM). **B,** the average effect of PAGly on AP-induced Ca^2+^ transient amplitude and SR Ca^2+^ load in the presence of propranolol (n = 5 myocytes). **C,** F/F_0_ profiles of cytosolic Ca^2+^ during AP- and caffeine-induced Ca^2+^ transients in control conditions, PKA inhibition with H89 (10 mM) with following application of PAGly (100 mM). **D,** the average effect of PAGly on AP-induced Ca^2+^ transient amplitude and SR Ca^2+^ load in the presence of H89 (n = 7 myocytes).

Next, we tested whether the stimulatory effect of PAGly on Ca^2+^ dynamics is mediated by β-adrenergic receptor activation. The treatment of cells with the β-adrenergic receptors inhibitor propranolol (10 μM) did not change the amplitude of AP-induced Ca^2+^ transients, however increased SR Ca^2+^ load to 129±30% (n=5 cells; Fig. 4A and B). Notably, the consequent addition of PAGly (100 μM) failed to induce any stimulatory effect on SR Ca^2+^ load and AP-induced Ca^2+^ transients compared to propranolol alone (Fig. 4B). β-adrenergic receptor activation stimulates adenylate cyclase and increases cytosolic cAMP level, which in turn stimulates PKA [9,11]. In the next experiments, we measured the effect of PAGly in the presence of the PKA inhibitor H89 (10 μM; Fig. 4C and D). H89 itself did not change the amplitude of AP-induced Ca^2+^ transients, but slightly increased SR Ca^2+^ load. Further treatment myocytes with PAGly (100 μM) failed to produce the stimulatory effects on AP-induced Ca^2+^ transients and SR Ca^2+^ load (Fig. 4C and D). Collectively, these results illustrate that PAGly invokes the stimulatory effect on intracellular Ca^2+^ dynamics by activating β-adrenergic receptors with the following activation of the PKA signaling cascade.

### The interaction between PAGly and ISO in regulation of Ca^2+^ signaling.

**Figure 5:**
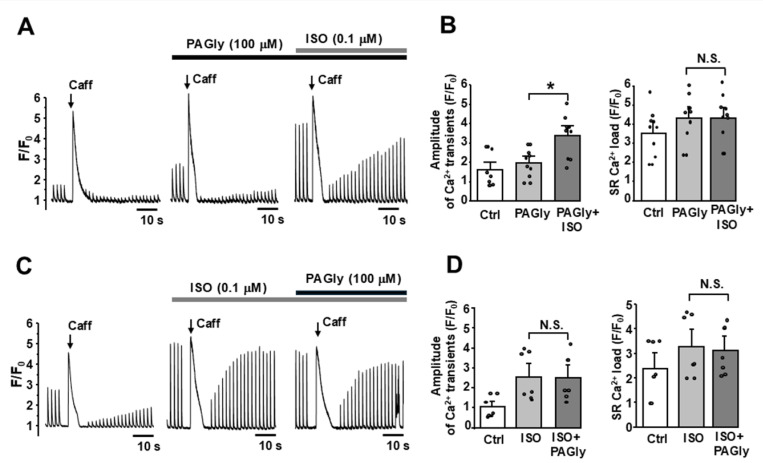
The competition between PAGly and ISO in regulation of Ca^2+^ signaling. **A,** F/F_0_ profiles of cytosolic Ca^2+^ during AP- and caffeine-induced Ca^2+^ transients in control conditions, during PAGly (100 mM) application and with following application of ISO (0.1 mM). **B,** The average effect of PAGly and PAGly + ISO on AP-induced Ca^2+^ transient amplitude and SR Ca^2+^ load (n = 10 myocytes). **C,** F/F_0_ profiles of cytosolic Ca^2+^ during AP- and caffeine-induced Ca^2+^ transients in control conditions, during ISO (0.1 mM) application and with following application of PAGly (100 mM). **D,** the average effect of ISO and ISO + PAGly AP-induced Ca^2+^ transient amplitude and SR Ca^2+^ load (n = 6 myocytes).

As our previous experiments showed that PAGly affects Ca^2+^ signaling by interacting with β-adrenergic receptors, we tested to what extent PAGly can compete with more selective adrenergic receptor agonist ISO. We analyzed the effects of ISO on intracellular Ca^2+^ signaling in cells pretreated with PAGly (Fig 5A). This analysis revealed that in the presence of PAGly (100 μM) the adrenergic agonist ISO (0.1 μM) increased the AP-induced Ca^2+^ transient amplitude to 172±31.3% (n=10 cells, Fig. 5B, left). Overall, the stimulatory effect of ISO on AP-induced Ca^2+^ transient was significantly smaller in the presence of PAGly: 170% ISO alone *vs.* 72% PAGly + ISO. ISO did not produce any further increase in SR Ca^2+^ load in the presence of PAGly (Fig. 5B, right). We also analyzed the effects of PAGly on Ca^2+^ regulation in myocytes pretreated with ISO (Fig. 5C). In this subset of experiments, ISO (0.1 μM) produced a strong stimulatory effect on Ca^2+^ regulation similar to those shown in Fig. 1C. The addition of PAGly (100 μM) in the presence of ISO (0.1 μM) did not produce any further increase of Ca^2+^ signaling as AP-induced Ca^2+^ transients and SR Ca^2+^ load remained the same as during ISO action alone (Fig. 5D). These data illustrate that PAGly affects Ca^2+^ signaling in cardiomyocytes by activating β-adrenergic receptors, but less efficiently than more selective adrenergic agonist ISO.

## DISCUSSION

It has been shown that the increased serum levels of the phenylalanine metabolites, such as PAGln and PAGly, positively correlate with the risk of developing cardiovascular diseases [2–4]. Analysis of PAGln level in patients at different stages of heart diseases suggests that PAGln is rather a causative factor of cardiomyopathy rather than a consequence of the disease [2,4,6,17,18]. There are several mechanisms through which PAGln and PAGly can contribute to the development of cardiac pathologies. For example, a high level of PAGln can facilitate transcriptions of genes associated with heart failure such as the B-type natriuretic peptide gene [4]. Moreover, PAGln and PAGly could affect the function of cardiovascular system by blunting adrenergic response during sympathetic stimulation [4,6,7]. This effect has been attributed to the competition between these phenylalanine metabolites and epinephrine for the binding to β-adrenergic receptors, suggesting that PAGln and PAGly could contribute to the blunted adrenergic response during the development of heart failure. It has been also proposed that PAGln and PAGly may act as an inhibitor of sympathetic stimulation, preventing the positive inotropic effect of epinephrine [4,6].

As heart contraction is critically dependent on well-controlled Ca^2+^ regulation [11], we investigated whether PAGly (more common phenylalanine metabolite in rodents) can also affect intracellular Ca^2+^ signaling in mice ventricular myocytes. We found that PAGly is more likely to increase intracellular Ca^2+^ dynamics by stimulating β-adrenergic receptors, leading to activation of the PKA-dependent signaling cascade (Fig 4). However, the stimulatory effect of PAGly on AP-induced Ca^2+^ transiens was significantly smaller than more selective adrenergic receptor agonist isoproterenol or ISO. We found that both PAGly and ISO produced a relatively similar increase in SR Ca^2+^ load (Fig 1 and 2). While ISO produced a strong increase in LTCC-mediated Ca^2+^ transient amplitude (Fig 3), surprisingly PAGly did not increase this component of Ca^2+^ signaling (Fig 3). As the cytosolic AP-induced Ca^2+^ transient is composed from both LTCC-mediated Ca^2+^ influx and SR Ca^2+^ release, this can explain the fact that PAGly had a smaller stimulatory effect on AP-induced Ca^2+^ transient amplitude than ISO. It appears that PAGly and ISO produced a different degree of stimulation of Ca^2+^ signaling in ventricular myocytes even though both compounds activate the β-adrenergic signaling cascade.

Our studies also revealed that ISO can induce the stimulatory response in myocytes pre-treated with PAGly, although smaller than those produced by ISO alone (Fig 5A and B). On the other hand, PAGly was ineffective to alter Ca^2+^ transients in myocytes treated with ISO (Fig 5 C and D). Therefore, it seems that the previously reported effect of PAGly on the contractility [4,7] is not linked to PAGly blocking the adrenergic cascade, but rather to other mechanisms. For instance, as membrane permeable metabolite PAGly could have direct interaction and negative effects on the contractile apparatus. Another explanation for a different response by PAGly and ISO is that these two molecules bind to different pools of adrenergic receptors. ISO selectively activates β1 and β2, which are the most abundant receptors expressed in cardiomyocytes, and they are most important for increasing myocardial contractility. On the other hand, PAGly binds with higher affinity to β2 and other receptors such as α2A, α2B [4,6,7]. These α receptors are also expressed in cardiomyocytes, but at lower levels compared to β1 and β2 [9,19]. Since PAGly can bind to different adrenergic receptors than ISO, it can still produce the stimulatory effect but is less efficient than ISO. Another explanation for the reduced effects of PAGly on Ca^2+^ signaling is that PAGly can bind and activate β1 adrenergic receptors, however with lower affinity than ISO. Since the affinity of ISO for β1 adrenergic receptors are higher than PAGly [6,19], ISO binding to β1 adrenergic receptors would be favored, overriding the PAGly effects. This explains why PAGly was ineffective to increase Ca^2+^ transients in the presence of ISO (Fig 5C). On the other hand, by competing for binding to β1 adrenergic receptors, PAGly can partially blunt the stimulatory effect of more selective β1 adrenergic receptor agonists such as ISO.

Overall, this study revealed that the gut microbiota metabolite PAGly can produce the stimulatory effect on Ca^2+^ signaling in mouse ventricular myocytes. This effect is largely mediated by β-adrenergic receptor stimulation and PKA activation. This, in turn, will phosphorylate the SERCA2a inhibitor PLB, causing Ca^2+^ pump activation, increases in SR Ca^2+^ load and in Ca^2+^ transients. However, high PAGly level can reduce the inotropic effect of adrenergic receptor activation by competing for β-adrenergic receptors with selective adrenergic agonists. Moreover, the chronic presence of PAGly in serum would reduce the adrenergic receptor response by causing desensitization of adrenergic receptors and blunting the adrenergic response.

## Data Availability

The data supporting this article and other findings are available within the manuscript, figures, and from the corresponding authors upon request.
